# Birth outcomes of individuals who have experienced incarceration during pregnancy

**DOI:** 10.1038/s41372-024-02170-4

**Published:** 2024-11-13

**Authors:** Emma Rose Miller-Bedell, Lillian Sie, Suzan L. Carmichael, Nana Matoba, Ya’el Weiner, Joseph J. Kim, Arash Anoshiravani, Dominika Seidman, Deirdre J. Lyell, Henry C. Lee

**Affiliations:** 1 Department of Pediatrics, Stanford School of Medicine, Stanford, CA USA; 2 Department of Obstetrics and Gynecology, Stanford School of Medicine, Stanford, CA USA; 3 Department of Pediatrics, University of California San Diego, La Jolla, CA USA; 4 Department of Obstetrics & Gynecology, University of California San Francisco, San Francisco, CA USA

**Keywords:** Epidemiology, Risk factors

## Abstract

**Objectives:**

Describe the prevalence, health, and birth outcomes of incarcerated pregnant individuals in California between 2011 and 2015.

**Study design:**

A population-based cohort study was performed using linked birth certificate and hospital discharge data. Associations between incarceration and birth outcomes were examined, including multivariable logistic regression to estimate odds ratios and 95% confidence intervals.

**Results:**

Amongst 1401 incarcerated and 551,029 nonincarcerated pregnant people across 112 delivery hospitals, 33% of incarcerated individuals had late initiation of prenatal care; 2.4% experienced severe maternal morbidity, compared to 18.9% and 1.6% of controls, respectively (*p* < 0.05). Births to incarcerated individuals had higher adjusted likelihoods of prematurity (OR 1.42, 95% CI 1.21, 1.67), small for gestational age (OR 1.31, 95% CI 1.11, 1.56), and NICU admission (OR 1.64, 95% CI, 1.40, 1.93) relative to controls.

**Conclusion:**

Incarcerated individuals have greater likelihood of negative birth outcomes. Identification of approaches to reduce these harms is warranted.

## Introduction

In 2022, 87,800 women over 18 years of age were incarcerated in state and federal prisons in the United States [1], and even more, 92,900, were incarcerated in city and county jails in the first half of 2022 alone [2]. The number of incarcerated females increased almost 5% from the end of 2021 to the end of 2022, but decreased by 19% compared to 2012 [3]. The majority were of childbearing age, between 18 and 44 years [1, 2]. Prior studies have estimated that 3-4% of women in prison, and 3–5% of women in jail are pregnant at intake [4, 5], but an accurate number remains unknown. Limited data are available on birth outcomes of incarcerated pregnant individuals, in part because such outcomes historically were not regularly published by government agencies such as the Bureau of Justice Statistics. Since the introduction of the First Step Act of 2018, the Bureau of Justice Statistics is newly mandated to provide yearly reports on birth outcomes in federal prison facilities; however, there remains a dearth of information on conditions and outcomes for pregnant individuals who enter into state prisons and jails [6].

Previous research on birth outcomes of those in state prisons and jails has presented mixed results. Some studies showed that incarceration may have a protective effect on birth outcomes, especially when incarcerated mothers are compared to at-risk controls [7–12]. It is possible that incarceration affords a standard level of nutrition, place to sleep, and presumed access to healthcare. A study conducted by the Bureau of Justice Statistics [13] interviewed prison and jail respondents, and reported that in general, sites provided pregnant individuals opportunity to provide a medical history and undergo screening tests, although many limitations were also highlighted that led to decreased access to and continuity in care. Some studies have found that incarceration has deleterious effects on birth outcomes such as infant mortality [14], birthweight, and prematurity [15, 16]. Such variability is unsurprising. Birth outcomes vary by state [5] and the healthcare and psychosocial support for pregnant mothers vary between different carceral facilities even within the same state, including access to sufficient nutrition, safe sleep arrangements, and policies such as shackling women during labor [17, 18]. In a prospective study of 6 U.S. jails, preterm birth rates ranged from 0 to 20%, with at least two reporting rates that were double the national rate, suggesting that individual systems contribute to variability [19].

It remains difficult to draw definitive conclusions about the impact of incarceration on birth outcomes. Our study aims to fill these gaps by evaluating birth outcomes of a large cohort of women who were incarcerated (any state or federal prison or jail) in California from 2011 to 2015.

## Methods

A retrospective population-based cohort study was performed using linked California birth certificate and hospital discharge diagnosis data from the California Department of Health Care Access and Information for the period January 1, 2011 to October 14, 2015 before the coding system changed from ICD-9 to ICD-10. Data were limited to ICD-9 as there may be differences in diagnosis and procedure code definitions between the two systems. Incarcerated pregnant individuals were defined as patients who have “Prison/Jail” listed as the site of admission or the site of discharge on their birth hospitalization record as reported to the state, or patients who delivered an infant with “Prison/Jail” listed as the site of discharge on the infant’s record. Because hospitals in California are required to report on the admission and discharge status of all patients to the state, information on the incarceration status of patients is likely to be more accurate than states that do not have a mechanism set up to collect these data. According to a report on prison nursery programs in California, almost all infants eligible for these programs were born to mothers who were incarcerated during pregnancy [20]. In this context, cases where the infant was coded as discharged to prison but whose mothers were not, were still included. Diagnosis codes, procedure codes and other information from the birth certificate were derived from hospital records. Deliveries were limited to singletons or the first infant record of multiple births (e.g., twins) to avoid repeat observations per pregnancy. Only records with information available for both the mother and infant were included in the analysis. We excluded hospitals with fewer than 5 births to incarcerated people, which resulted in excluding 61 hospitals and 132 births to incarcerated mothers. The purpose of this exclusion was to minimize the possibility of bias given that not all hospitals may consistently code for discharge to and admission from prison. We also considered that hospitals that cared for no or few individuals affected by incarceration may have very different hospital and population characteristics compared to included hospitals and have unmeasured confounders that could strongly influence outcomes.

Neonatal and maternal outcomes were selected based on prior literature that examined associations of incarceration and pregnancy. Predictor variables included both social and medical risk factors including those that may be related to nutrition and adequacy of pregnancy care. Variables were derived from ICD-9 codes in the hospital records and from birth certificate data. Maternal infection was indicated by ICD-9 code 647.9. Maternal death was excluded as an outcome variable, as it was too rare according to state guidelines for reporting.

Bivariate analysis was conducted with chi-square and Fisher exact tests; *p*-values were calculated with two-sided tests. Associations between incarceration and selected maternal and neonatal outcomes were examined with multivariable logistic regression using the Firth penalized likelihood method to account for small cell sizes. Potential confounding factors were adjusted in the multivariate analysis. Twenty-four records were excluded from multivariable analysis due to missing maternal age. Analyses were conducted using SAS 9.4 (SAS Institute, Cary, NC, USA). The study was approved by the California Committee for the Protection of Human Subjects and the Stanford University Institutional Review Board. Informed consent was waived because of minimal risk. This research was conducted in accordance with the Declaration of Helsinki. Data used for this study are not publicly available because of patient privacy. Institutions wishing to access this data may submit a request form to the California Department of Health Care Access and Information [21].

## Results

Our results included 1401 births to pregnant individuals who were incarcerated and 551,029 nonincarcerated pregnant controls across 112 hospitals in California.

The majority of incarcerated and non-incarcerated people were between the ages of 20–40 (Table 1). Forty-six percent of the incarcerated population was Hispanic, 27% non-Hispanic White, 17% non-Hispanic Black, 3% Asian/Pacific Islander, 2% American Indian/Alaskan Native (AI/AN), and 5% Other (Table 1). The most overrepresented racial and ethnic groups of incarcerated individuals compared to controls were AI/AN, non-Hispanic Black, and non-Hispanic White patients (Table 1). Compared to non-incarcerated controls, incarcerated individuals had higher rates of prenatal care paid by the government (83% vs. 67%) and lower rates of having a high school diploma (55% vs. 71%) or graduate degree (2% vs. 17%) (Table 1).

**Table 1:** Demographic information for incarcerated and control populations.

	Incarcerated population (*N* = 1401)	Control population (*N* = 551,029)	*P*-Value
**Mother Age**			<0.0001
<20	60 (4.3)	46,926 (8.5)	
20–34	1146 (81.8)	415,413 (75.4)	
35–40	168 (12.0)	76,383 (13.9)	
>40 or no information^a^	27 (1.9)	12,307 (2.2)	
**Mother Race/Ethnicity**			<0.0001
Hispanic	615 (45.7)	341,819 (64.4)	
Non-Hispanic White	368 (27.4)	104,079 (19.6)	
Non-Hispanic Black	229 (17.0)	26,280 (5.0)	
Asian/Pacific Islander	36 (2.7)	41,067 (7.7)	
American Indian /Alaska Native	24 (1.8)	1568 (0.3)	
Other	73 (5.4)	16,210 (3.1)	
No Information	56 (.)^b^	20,006 (.)^b^	
**Education**			<0.0001
No High School diploma	587 (45.1)	149,844 (29.0)	
High School Graduate	469 (36.0)	150,701 (29.2)	
Associate degree or some college	218 (16.7)	129,871 (25.1)	
College graduate	29 (2.2)	86,122 (16.7)	
No Information	98 (.)^b^	34,491 (.)^b^	
**Principle payer source for prenatal care**			<0.0001
Private	51 (3.6)	152,871 (27.7)	
Government^c^	1161 (82.9)	369,206 (67.0)	
Self Pay or Other	92 (6.6)	20,887 (3.8)	
No Prenatal Care or Unknown Payer Source	97 (6.9)	8065 (1.5)	

^a^The categories for “maternal age >40” and subjects with “no information available” were combined to mask small cell size to protect patient privacy.

^b^Percentages for missing data are not calculated, not reported and is represented as “.”

^c^Government payer source includes Medi-Cal, Title V maternal and child health grants, medically indigent services and other government programs at the federal, state and local levels.

Percentage of total in parentheses.

Eighty-one percent of incarcerated individuals had one or more prior children compared to 64% of non-incarcerated controls (Table 2). We found significantly higher rates of infections affecting pregnancy (*p* < 0.01) among incarcerated individuals (Table 2). There were no significant differences between groups in the occurrence of preeclampsia, prolonged rupture of membranes, or gestational hypertension (*p* > 0.05) (Table 2). Incarcerated individuals had significantly higher rates of substance use (3% vs. 0.2% *p* < 0.01) and mental health disorders (31% vs. 5%, *p* < 0.01) (Table 2). Thirty-three percent of incarcerated individuals had initiation of prenatal care in the second or third trimester compared to 19% of controls and had significantly higher rates of severe maternal morbidity (2.4 vs. 1.6%, *p* < 0.05) (Table 2).

**Table 2:** Maternal health characteristics and birth outcomes of incarcerated and control populations.

	Incarcerated population *N* = 1401	Control population *N* = 551 029	*P*-value
**Number of prior children**			<0.0001
1	295 (21.1)	166,900 (30.3)	
2 to 3	512 (36.6)	152,800 (27.7)	
4 or more	331 (23.6)	34,475 (6.3)	
0	263 (18.8)	196,854 (35.7)	
**Prenatal care onset time**			<0.0001
1st trimester	818 (58.4)	431,263 (78.3)	
2nd trimester	329 (23.5)	83,014 (15.1)	
3rd trimester	127 (9.1)	20,834 (3.8)	
No prenatal care or unknown timing of prenatal care onset	127 (9.1)	15,918 (2.9)	
**Mode of delivery**			<0.0001
Vaginal - spontaneous	851 (60.7)	348,488 (63.2)	
Vaginal - operative	24 (1.7)	18,590 (3.4)	
Cesarean - primary	189 (13.5)	79,007 (14.3)	
Cesarean - repeat	317 (22.6)	90,441 (16.4)	
Cesarean - primary or repeat with failed trial of labor	20 (1.4)	14,503 (2.6)	
**Other conditions**			
Hypertension - gestational	34 (2.4)	13,358 (2.4)	0.99
Preeclampsia	34 (2.4)	13,308 (2.4)	0.98
Prolonged rupture of membranes	34 (2.4)	13,285 (2.4)	0.97
Maternal infection	134 (9.6)	15,922 (2.9)	<0.0001
Placental abruption	29 (2.1)	5804 (1.1)	0.0002
Diabetes - gestational	124 (8.9)	64,317 (11.7)	0.0010
Diabetes - preexisting	18 (1.3)	8101 (1.5)	0.56
Hypertension - preexisting	191 (13.6)	58,157 (10.6)	0.0002
Obesity	275 (19.6)	144,223 (26.2)	<0.0001
Asthma	112 (8.0)	16,218 (2.9)	<0.0001
Mental Health Disorders^a^	435 (31.1)	24,923 (4.5)	<0.0001
Smoking	199 (14.2)	14,105 (2.6)	<0.0001
Drug Dependence	48 (3.4)	1193 (0.2)	<0.0001
Severe Maternal Morbidity	33 (2.4)	8954 (1.6)	0.03
Low Birthweight	120 (8.6)	36,730 (6.7)	0.0044
Very Low Birthweight	22 (1.6)	6976 (1.3)	0.31
NICU Admissions	175 (12.5)	43,050 (7.8)	<0.0001
**Gestational age**			<0.001
31 weeks or less	25 (1.8)	7840 (1.4)	
32–36 weeks	153 (10.9)	39,984 (7.3)	
37–40 weeks	1207 (86.2)	501,797 (91.1)	
> 40 weeks or Unknown	27 (1.9)	12,307 (2.2)	

Percentage of total in parentheses.

^a^Mental health disorders include: intellectual disabilities; mental disorders complicating pregnancy; neurotic disorders, personality disorders, and other mental disorders; organic psychotic conditions; and, other psychoses.

Babies born to incarcerated individuals had significantly higher odds of prematurity (GA < 37 weeks) (OR 1.42; 95% CI 1.21 to 1.67), small for gestational age (OR 1.31; 95% CI 1.11 to 1.56), and NICU admission (OR 1.64; 95% CI 1.40 to 1.93) after adjusting for maternal age, multiple gestation, parity, smoking, and body mass index (Table 3). The prevalence of very low birthweight (OR 1.28; 95% CI 0.84 to 1.96) was not significantly different between the two populations (Table 3).

**Table 3:** Odds ratio of birth outcomes for incarcerated compared to non-incarcerated individuals, estimated from multivariable logistic regression models adjusted for maternal age, multiple gestation, parity, smoking, and BMI.

Effect	Odds Ratio Estimate	Lower 95% Confidence Limit	Upper 95% Confidence Limit
Gestational Age <32 weeks	1.24	0.83	1.85
Gestational Age <34 weeks	1.56	1.19	2.05
Gestational Age <37 weeks	1.42	1.21	1.67
NICU admission	1.64	1.40	1.93
Low Birthweight	1.27	1.05	1.54
Very Low Birthweight	1.28	0.84	1.96
Small for Gestational Age	1.31	1.11	1.56
Large for Gestational Age	0.89	0.74	1.08

## Discussion

We found adverse associations in health outcomes for individuals who were incarcerated either directly before or upon hospital discharge from giving birth. Further, babies born to this population had higher rates of negative health outcomes that make them higher risk for both immediate and long-term complications.

In contrast to studies from a single facility, we included hospitals from across California, enhancing generalizability to the broad pregnant population affected by incarceration. This is an advantage over prior studies [12, 15, 22] that draw conclusions about birth outcomes from a single carceral facility making it difficult to generalize to the incarcerated population as a whole, in part because perinatal care varies between individual facilities and states. Prior ecological studies have examined regional incarceration rates and pregnancy outcomes or examined parental incarceration and outcomes, but have been limited by not accounting specifically for individual maternal incarceration [16, 23, 24].

The proportion of non-Hispanic Black people in the incarcerated population was 3.4 times greater than the non-incarcerated control population (17.0% vs. 5.0%), similar to the disparity in incarceration rates among non-Hispanic black people versus other groups seen in the general population [25]. The AI/AN racial group accounted for 1.8% of births in the incarcerated sample, which was 6 times greater than their representation among non-incarcerated controls and 4.5 times greater than the California AI/AN proportion of births (1.8% vs. 0.3% vs. 0.4%) [25]. The overrepresentation of births affected by incarceration to individuals who are Black and AI/AN reinforces other aspects of structural racism embedded in our legal system. Incarcerated individuals had higher rates of prenatal care paid for by the government and lower rates of higher education compared to non-incarcerated controls, which is unsurprising given the lives of many are afflicted with poverty and limited access to healthcare [5, 26, 27].

The roots of unequal incarceration of Black, indigenous, and economically disadvantaged people have been well described. The racial disparity in mass incarceration has been compared to slavery (1619–1865), the Jim Crow segregation in the South (1865–1965), and the urban ghetto in the North (1915–1968) [28]. The collateral damage of mass incarceration to Black / African American communities includes damage to social networks, distortion of social norms, and destruction of social citizenship [29]. As Dorothy Roberts noted, “we need to reconsider the meaning of reproductive liberty to take into account its relationship to racial oppression” [30].

Previous research shows parental incarceration is associated with child health problems such as asthma, migraines, high cholesterol, early grade retention, depression, anxiety, post-traumatic stress disorder, antisocial behavior, human immunodeficiency virus / acquired immunodeficiency deficiency syndrome, and poor health [31–34]. Health problems such as increased likelihood of worse mental and physical health extend into adulthood [35, 36]. We found that 81% of pregnant incarcerated individuals had prior children, which is higher than a prior Bureau of Justice Statistics report that found that 58% of females in prisons across the United States have minor children; this may indicate that some pregnant individuals affected by incarceration have older children who are no longer minors [37]. Prior to the COVID-19 pandemic, there were approximately 5 million U.S. children with at least one parent in prison at one time or another [38], and 77% of mothers in state prison who had lived with their children just prior to incarceration provided most of the children’s daily care [4]. The high frequency of incarcerated parents with children reminds us of the multigenerational ramifications of incarceration during pregnancy.

Individuals in prison or jail are more likely to have ever had a chronic condition, an infectious disease, a cognitive disability, or any disability [39, 40] consistent with our finding that compared to non-incarcerated controls, the rate of severe maternal morbidity was increased 1.4 times, the rate of placental abruption nearly double, and the rate of infections more than three times increased. While our findings suggest that incarcerated individuals have an increased likelihood of complex medical needs, our study troublingly found they were more likely to have later onset of prenatal care compared to controls, with one-third of incarcerated pregnant people initiating care in either the 2nd or 3rd trimesters. Such delay in care speaks to the instability of healthcare of individuals affected by incarceration, some of whom may enter into the carceral system, especially jail where the average length of stay is 26 days [2] and leave before childbirth but may still have deleterious effects from the experience [26].

The incarcerated population is much more socially at risk for poverty and other adverse life circumstances, with significant pediatric ramifications. Maternal perinatal depression and anxiety alone can lead to adverse childhood experience, and is associated with preterm birth, low birthweight, intrauterine growth restriction, as well as impaired social interaction, and delays in language, cognitive, and social-emotional development [41]. The developmental outcomes extend beyond infancy into childhood and adolescence [41, 42]. Complications from these conditions can have enduring ramifications for not only the birthing parent but also the offspring and entire family unit [43, 44]. Compared to non-incarcerated pregnant controls, the rate of mental health disorder was 6.9 times higher and substance use was 15.6 times higher in the incarcerated pregnant group. The ramifications for the immediate perinatal health of the birthing parent can be severe. A report from nine maternal mortality review committees found that mental health conditions and substance use disorders (SUDs) were linked to 12.9% and 8.2% of pregnancy-related deaths, respectively [45]. As discussed by Haffajee et al. [44], the number of states that have adapted punitive policies (including fines, years of jail time, and loss of custody) directed at pregnant individuals with SUDs has increased from 12 states in 2000 to 26 states in 2017. In California, mental health disorders and substance use are screened at intake, with follow-up evaluations and referrals made available to those in need for treatment. Policies and programs that can facilitate rehabilitation and treatment as an alternative to punitive measures for those with SUDs may help to optimize the health for both the pregnant individual and their child.

We found significantly higher rates of preterm births of babies born to incarcerated mothers than controls, where 87% of babies born to incarcerated mothers were term compared to 91% of babies in the control population. The rates for both populations exceeded California’s preterm birth rate of 8.5% in 2015 and the nation’s preterm birth rate of 9.85% in 2016 [25], which may reflect the nature of hospitals that tend to care for incarcerated individuals. We also observed higher rates of small for gestational age and low birth weight in babies born to incarcerated individuals than controls. In addition, we found significantly higher rates of NICU admission, reaching 13% for babies born to incarcerated mothers compared to 8% for controls. The discrepant rates of preterm births, small for gestational age, and NICU admissions persisted even when correcting for maternal age, multiple gestation, parity, smoking, and body mass index. We did not observe a significant difference in very low birthweight.

Findings from our study indicate incarceration has negative ramifications for birth outcomes and stands in contrast to other studies [7–12]. In fact, some prior studies have shown that incarceration has a protective effect on birth outcomes, especially when incarcerated mothers are compared to at-risk controls, such as non-jailed methadone-maintained women [10], women living in “high-risk” residential areas [12], or people with history of criminal conviction or drug use [9, 11], leading to speculation that incarceration may provide protective effects on pregnancy by providing shelter, food, healthcare, and reduction of substance misuse [7, 8]. Those prior studies indicate that directed efforts to optimize pregnancy healthcare during incarceration can have a positive impact. The population affected by incarceration had significantly higher rates of medical morbidities, mental health disorders and drug dependence (Table 2), indicating that public health investment in optimizing healthcare in these areas outside of the carceral system, may mitigate risk for these individuals.

The increased rates of maternal morbidities among women who are incarcerated have been previously described. Using the National Inpatient Sample from 2015 to 1018, Logue et al. [46] found that pregnancy complications associated with incarceration included non-transfusion severe maternal morbidity, hypertensive disorders of pregnancy, preterm delivery, placental abruption and postpartum hemorrhage. Similar to our findings, they found increased abruption among incarcerated pregnant women, and because the associations remained significant after adjustment for confounders, it is suggested that some contribution to maternal risk is conferred by incarceration. This study would not have had linkage to the vital records as in our study.

We excluded 61 California hospitals in order to minimize the possible bias encountered by hospitals that do not routinely admit incarcerated pregnant individuals. It is possible that this could lead to clusters of negative birth outcomes in selection bias. It is also possible that individual differences in risks of adverse birth outcomes, such as timing and duration of incarceration during pregnancy, play a role, but are not captured in administrative datasets. Finally, our findings may be related to the control population, a statewide population of non-incarcerated women in California. The 9.1% preterm birth rate in California ranks lower than most states and may have provided a control group healthier than other studies [47], some of which use control groups of populations similarly disadvantaged to incarcerated individuals, such as women who were incarcerated during the same time but not pregnant [48].

Scholars such as Angela Davis, Mariame Kaba, and Ruth Wilson Gilmore have stated that the incarceration system is designed to punish and dehumanize people [49–51]. Comprehensive progress may come from shifting away from investing in reforming our current carceral system and instead reimagining the justice system through centering the voices of people most impacted by incarceration, through rehabilitation and restoration, and through investing in underserved communities. In 2021, the Minnesota government enacted the Healthy Start Act that allows individuals who are serving short sentences in state prison to be released conditionally for the duration of their pregnancy up to the first year after birth [52].

Our study had several limitations. The carceral system in California may differ from other states. While California does not have prisons specifically designated for pregnant people, as many as 17 states have one specific facility that houses incarcerated pregnant individuals [5]. Female incarceration rates vary by state, with Texas having 178 women incarcerated per 100,000 residents, compared to 88 per 100,000 in California [53]. The dataset we used does not distinguish between facilities (i.e. jail, state, and federal prisons) and the exact duration and time of incarceration in relation to pregnancy is unknown. The disposition variable to determine admission to or discharge from the hospital in relation to the carceral system may not be well coded in healthcare facilities. We worked within the constraints of using birth certificate data and hospital discharge data, recognizing that both have limitations and are sometimes discordant; our prior experiences informed the study methods [54, 55]. Fetal birth certificate data were not included so pregnancies that ended before 20 weeks of gestation were not captured in this study. We could not definitively determine whether some maternal comorbidities, such as obesity, were present during the current pregnancy or if they were from an individual’s past medical history. The size of the dataset makes correcting for some confounding variables infeasible.

## Conclusion

We describe negative effects of incarceration on the current pregnancy and racial/ethnic disparities in who is incarcerated during pregnancy. We found that the incarcerated pregnant population is at higher risk for poor pregnancy outcome; it is not known if this is due to the type and effect of incarceration. The findings of our study suggest the importance of future work to mitigate such outcomes and their impact on both the pregnant individual and their baby.

## Data Availability

The data that support the findings of this study are available from the state of California but restrictions apply to the availability of these data, which the investigators were given permission to use for this research, and so are not publicly available. Data can be requested from the state of the California Department of Health Care Access and Information.
